# Application of Percutaneous Needle Electrolysis Does Not Elicit Temperature Changes: An In Vitro Cadaveric Study

**DOI:** 10.3390/ijerph192315738

**Published:** 2022-11-26

**Authors:** Sergio Borrella-Andrés, Miguel Malo-Urriés, Albert Pérez-Bellmunt, José L. Arias-Buría, Jacobo Rodríguez-Sanz, María Isabel Albarova-Corral, Vanessa González-Rueda, Gracia M. Gallego-Sendarrubias, César Fernández-de-las-Peñas, Carlos López-de-Celis

**Affiliations:** 1 Health Sciences Faculty, University of Zaragoza, 50009 Zaragoza, Spain; 2 Faculty of Medicine and Health Science, Universitat Internacional de Catalunya, 08017 Barcelona, Spain; 3 ACTIUM Functional Anatomy Group, Universitat Internacional de Catalunya, 08195 Sant Cugat del Vallès, Spain; 4 Department of Physical Therapy, Occupational Therapy, Rehabilitation and Physical Medicine, Universidad Rey Juan Carlos, 28922 Alcorcón, Spain; 5 Department of Physical Therapy, Fundació Institut Universitari per a la Recerca a l’Atenció Primària de Salut Jordi Gol i Gurina, 08007 Barcelona, Spain; 6 Department of Physical Therapy, Camilo José Cela University, 28692 Madrid, Spain

**Keywords:** percutaneous needle electrolysis, temperature, EPI, cadaver

## Abstract

Percutaneous needle electrolysis (PNE) consists of the ultrasound-guided application of a galvanic electrical current through a solid filament needle. One proposed therapeutic mechanism for this intervention is a potential thermal effect. The aim of this study was to investigate if the application of PNE induces changes in temperature in different cadaveric musculoskeletal tissues. A repeated measure experimental cadaveric study was designed with 10 cryopreserved knees (5 men, 5 women). Sterile stainless-steel needles of 40 mm length and 0.30 mm caliber were used in this study. An ultrasound-guided needling puncture was performed in the targeted tissue (patellar tendon, infra-patellar fat, and vastus medialis muscle). Additionally, the tip of the needle was placed next to the thermometer sensor at the minimum possible distance without direct contact with it. The temperature differences before and after different applications were measured. The applications were: three applications for 3 s of 3 mA of intensity (3:3:3) when the tendon was the targeted tissue, three applications for 3 s of 1.5 mA of intensity (1.5:3:3) when the fat or muscle was the targeted tissue, and 24 s of 1 mA of intensity (1:24:1) in all tissues. No statistically significant Group*Time interactions were found in any tissue (tendon: F = 0.571, *p* = 0.459, ŋ^2^ = 0.03; fat pad: F = 0.093; *p* = 0.764, ŋ^2^ = 0.01; muscle: F = 0.681; *p* = 0.420, ŋ^2^ = 0.04). Overall, no changes in temperature were observed between both applications in the tendon (3:3:3 vs. 1:24:1) and fat/muscle (1.5:3:3 vs. 1:24:1) tissues. The application of two different percutaneous needle electrolysis protocols did not produce appreciable thermal changes in the tendon, fat, and muscle tissues of human cadavers. The results from the current cadaver study support that a thermal effect should not be considered as a mechanism of clinical action regardless of the targeted human tissue when applying percutaneous needle electrolysis since no changes in temperature after its application were observed.

## 1. Introduction

Percutaneous needle electrolysis (PNE) consists of the ultrasound-guided application of a galvanic electrical current through a solid filament needle [1]. It has been found that PNE produces an inflammatory reaction in the treated tissue [2] and a marked increase in pH [basic] at the tip of the needle, which could hydrolyze scar tissue [3,4]. Electrolysis is defined as a process by which water (H_2_O) and sodium chloride (NaCl), which are found in the human tissues, are broken down into their constituent chemical elements and quickly regroup to form completely new substances, sodium hydroxide (NaOH), dihydrogen (H_2_), and dichloride (Cl_2_), as a consequence of the passage of a continuous electric current flow [5]. Using the cathode as an active electrode, PNE is able to create a change in pH and an increase in the partial pressure of oxygen, allowing phagocytosis and tissue repair [6]. The production of an inflammatory response is focused on reestablishing tissue repair on the cathode and has been attributed to the increase in NaOH, resulting from the electrolysis produced.

PNE is used as an electrochemical intervention, inducing cell necrosis throughout the electrolytic reaction produced by the electrical current flow through the needle [1]. This procedure generates a controlled inflammatory response in a specific targeted tissue, allowing phagocytosis of degenerated tissue, permitting a specific posterior repair [4]. The clinical effects of PNE have been evaluated when applied to different tissues such as tendons [7,8], muscles [9], or nerves [3,4]. In fact, a recent meta-analysis has found moderate-quality evidence supporting a positive effect of ultrasound-guided PNE for decreasing pain and related disability in musculoskeletal pain conditions [10].

Despite inducing an inflammatory response in the tissue and a local temperature increase in the area of application as measured by infrared thermography [11], PNE is considered a non-thermal intervention. Among those studies conducted to date, only one has verified the non-thermal effect of PNE by applying galvanic current in a Ringer’s saline solution and controlling the temperature changes of the solution using a digital thermometer [12]. No evidence is available on temperature changes after the application of PNE in human musculoskeletal tissues (i.e., muscle, tendon, or fat) where the tissue resistance to the passage of the electrical current could induce an increase in temperature.

Studying temperature changes produced by the PNE is of great interest since the increase in temperature on a small caliber needle could put at risk the integrity of the tissue/structure on which the intervention is applied and surrounding important structures, such as arteries, veins, or nerves. In the same way, the confirmation of this intervention as a non-thermal approach contributes as an advance in the knowledge of its underlying mechanisms, being able to completely rule out the thermal effect, providing more weight and relevance to the mechanical effect of the needle and the electrochemical effect of the galvanic electrical current. Accordingly, the objective of the current cadaveric study was to investigate if the application of the PNE induces changes in temperature in different musculoskeletal tissues.

## 2. Methods

### 2.1. Study Design

A repeated measure experimental cadaveric study was designed to establish the effects of PNE on temperature in different knee tissues: vastus medialis, patellar tendon, and infrapatellar fat pad. The body donation program of the anatomy laboratory of the University of Catalunya provided all the samples. The study was approved by the local committee (reference number CBAS-2021-10).

### 2.2. Cadaveric Specimens

The sample was composed of 10 cryopreserved knees (5 men, 5 women), aged 67 to 85 years. None of the cadaveric samples used in this study had evidence of trauma or surgical scars on the limbs. The frozen samples were stored at −20 °C and were thawed at room temperature 24 h prior to the experiment.

### 2.3. Temperature Assessment

All instrumentation used in this study possessed a calibration certificate. Before performing the needling insertion, the thermometer sensor “Hart Scientific PT25 5628-15” (ICC > 0.97, standard error of measurement < 0.03 °C, minimum detectable change < 0.08) [13] was placed in the targeted tissue. A minimal incision was performed using a scalpel in the superficial planes to facilitate sensor placement. Once located in the superficial planes, it was ultrasound-guided (Logic eR8 General Electric) with a high-frequency linear transducer (L4-12T-RS) in the targeted tissue. The sensor and ultrasound probe remained stable during the intervention. This thermometer focuses the temperature assessment on the tip, as has been previously performed [14]. When the thermometer was introduced, a temperature stabilization time was allowed. The temperature was recorded before and after each application of PNE on each tissue. A 1 min rest period was considered between applications.

### 2.4. Needling Procedure

Sterile stainless-steel needles with a cylindrical plastic guide, of 40 mm length and 0.30 mm caliber (AguPunt EPI^®^) were used in this study. A “clean technique”, including hand washing, sterile latex-free exam gloves, and cleaning the skin with an alcohol swab prior to the application, was conducted for mimicking real clinical practice [15].

**Figure 1:**
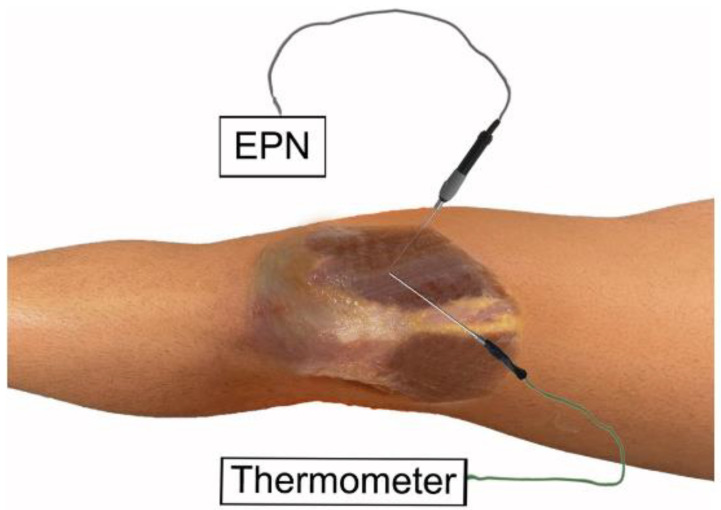
Percutaneous needle electrolysis on the vastus medialis on an anatomic sample.

**Figure 2:**
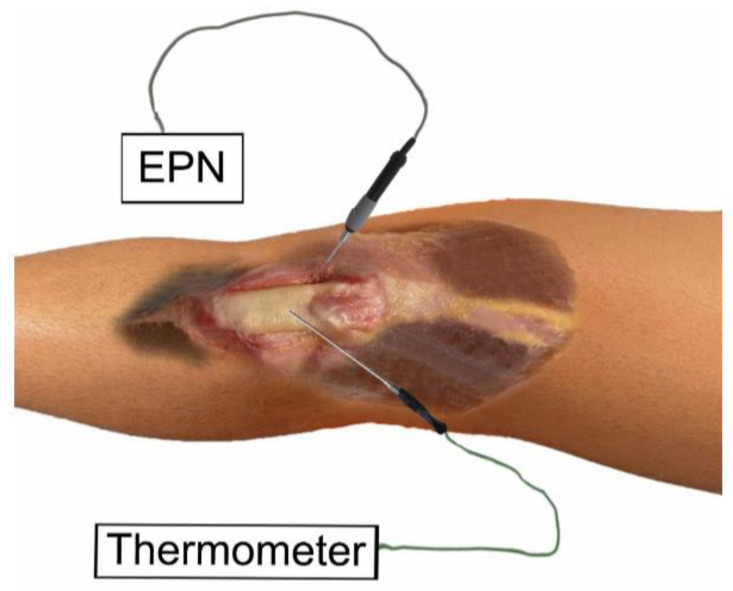
Application of percutaneous needle electrolysis on the patellar tendon on an anatomic sample.

**Figure 3:**
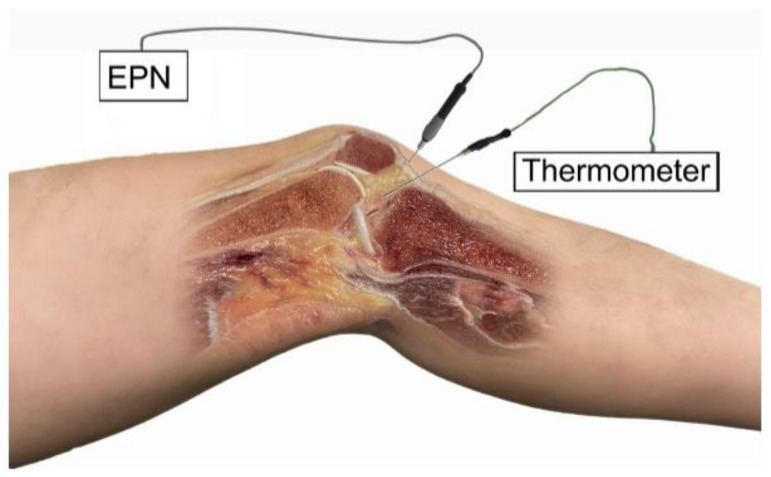
Application of percutaneous needle electrolysis on the infrapatellar fat pad of the knee on an anatomic sample.

The ultrasound-guided needling puncture was performed by a physical therapist with more than 10 years of experience. The needling puncture was performed guided by ultrasound, directing the needle from the opposite end of the probe, following the long axis, allowing the visualization of the path and the arrangement of the needle during the procedure, advancing until the tip of the needle reached the targeted tissue:

(A) Vastus medialis (Figure 1), the most medial muscle of the extensor apparatus of the knee, as it plays an important role in the function of the patella femoral joint [16].
(B) Patellar tendon (Figure 2), the single and straight tendon which connects the patella to the tibial tubercle, transmitting force generated by the quadriceps muscles onto the tibia [17].
(C) Infrapatellar fat pad (Figure 3), a fibro-adipose tissue, the site of insertion of the infrapatellar and medial synovial plicae [18].

Additionally, the tip of the needle was placed next to the thermometer sensor at the minimum possible distance without direct contact with it.

### 2.5. Percutaneous Needle Electrolysis Procedure

The PNE intervention was applied with the EPI^®^ Alpha CE0051 Equipment (EPI ADVANCED MEDICINE, Spain). The anode patch was placed onto the skin surface close to the knee, whereas the cathode needle was inserted into the target tissue (muscle, fat, or tendon). Two different commonly used protocols of PNE were studied. The first protocol (“intermittent application”) included three applications for 3 s of 3 mA of intensity (3:3:3) when the tendon was targeted or three applications for 3 s of 1.5 mA of intensity (1.5:3:3) when the fat or muscle was the targeted tissue. The second protocol (“continuous application”) consisted of an application for 24 s of 1 mA of intensity (1:24:1) in all tissues. This procedure was repeated ten times on each tissue for each protocol. Between applications, a 1 min rest period was given to allow the normalization of the temperature, until the thermometer did not oscillate the indicated temperature.

### 2.6. Statistical Analysis

Statistical analysis was performed using the SPSSv.20 statistical package. The Shapiro–Wilk test was used to assess the normal distribution of the variables. Means and standard deviations were expressed for all variables.

A linear mixed model (ANOVA) with time (baseline, post-intervention) as the within-subject variable and group (EPI-EPM) as the between-groups variable was conducted to determine the changes in all the outcomes. Effect sizes (ES) were calculated using eta squared (ŋ^2^). Considering an effect size > 0.14 as large, around 0.06 were medium and < 0.01 were small [19]. Statistical analysis was performed by intention-to-treat. The significance level was set at *p* < 0.05.

## 3. Results

Table 1 shows the values of temperature expressed in o C before and after applying different electrical current protocols on each tissue. The repeated measure ANOVA revealed no statistically significant Group*Time interactions in any tissue (tendon: F = 0.571, *p* = 0.459, ŋ 2 = 0.03; fat pad: F = 0.093; *p* = 0.764, ŋ 2 = 0.01; muscle: F = 0.681; *p* = 0.420, ŋ 2 = 0.04). Overall, no changes in temperature were observed between both applications in the tendon (3:3:3 vs. 1:24:1) and fat/muscle (1.5:3:3 vs. 1:24:1) tissues.

## 4. Discussion

This cadaveric study examined thermal changes in three different human tissues after the application of two different protocols of PNE. To the best of the author’s knowledge, this is the first in vitro study analyzing thermal changes produced by the application of PNE in different human tissues. According to Joule’s law, an electrical current that passes through a conductor produces an increase in temperature that depends on the intensity of the current applied, the resistance of the tissue, and the application time. This fact could explain different behaviors in the variation of temperature since fat is a poor conductor, tendon is a medium conductor, and muscle is a generator tissue [20,21].

The protocols evaluated ((3:3:3), (1.5:3:3), and (1:24:1)) in the current cadaveric study have been previously clinically used and found to be effective in the management of patellar tendinopathy, subacromial pain syndrome, whiplash syndrome [9], or temporomandibular myofascial pain [22]. We aimed to analyze if these protocols generated a thermal or non-thermal effect in a cadaver human tissue. Despite the different intensities and application times, we did not observe significant temperature variations in our study, suggesting that PNE does not produce temperature increases at the administered doses. This lack of temperature changes could be explained by the low doses and short periods of application used in clinical practice, or the fact that the type of tissue in which the PNE is applied does not seem to have any influence on the temperature changes produced. Accordingly, current results suggest that PNE should be considered as a thermally safe technique.

These results put more emphasis on the dosage, in agreement with a recent study in which the mechanism of action of PNE is questioned [23]. Previous explanations of tissue repair mechanisms for PNE were based on Na OH release and local pH alkalization [23]. However, a recent study has rejected this hypothesis, opening the option to other possible explanations, such as a potential inflammatory response mediated by NLRP3 inflammasome [24]. Galvanic current is also applied in high doses as electrolytic ablation by surgeons applying 100 times higher doses [25] than in PNE, doses which physiotherapy galvanic electronic devices cannot achieve. In fact, high-intensity doses for long exposure times or repeated high-intensity impacts can produce greater necrosis in the tissue, not associated with tissue recovery [25]. Therefore, high doses are not advisable for clinical practice, as has been previously demonstrated in a recent study [24]. Considering that temperature is not a differential or limiting factor for calculating the treatment dose since no changes are obtained, formulas to calculate the most appropriate dose for each type of human tissue according to the tissue damage are needed to reduce treatment variability. As a concluding remark, the current study reinforces the idea of there being no thermal effects of the application of galvanic electrical current using the PNE technique in human tendon, muscle, and fat tissues.

Finally, our results should be considered attending to the conditions of the study. First, the non-thermal effect can only be attributed to both protocols used and not to other types of applications of PNE. Protocols with higher doses or prolonged periods could be of interest to further determine their thermal effects. Second, although this was an in vitro study, we used cadaver tissue whose response could be slightly different from human living tissue.

## 5. Conclusions

The application of two different percutaneous needle electrolysis protocols did not produce appreciable temperature changes in the tendon, fat, and muscle tissues of human cadavers. These results support that a thermal effect should not be considered as a mechanism of clinical action regardless of the targeted human tissue when applying percutaneous needle electrolysis.

## Figures and Tables

**Table 1:** Changes in temperature over tendon, fat, and muscle before and after the application of percutaneous needling electrolysis.

	(3:3:3) ^1^/(1.5:3:3) ^2^	(1:24:1)
	Mean ± SD	Mean ± SD
**Tendon before (°C)**	31.2 ± 3.0	31.1 ± 1.2
**Tendon after (°C)**	31.2 ± 3.1	31.0 ± 1.3
**Fat before (°C)**	31.7 ± 2.6	31.1 ± 1.3
**Fat after (°C)**	31.7 ± 2.7	31.1 ± 1.3
**Muscle before (°C)**	31.5 ± 3.4	30.9 ± 1.1
**Muscle after (°C)**	31.9 ± 3.9	31.0 ± 1.2

^1^: First protocol tendon dosage, ^2^: First protocol muscle/fat dosage.

## Data Availability

Data are available upon request to the lead author.

## References

[1] Sánchez Ibañez J.M. (2009). Clinical Course in the Treatment of Chronic Patellar Tendinopathy through Ultrasound Guided Percutaneous Electrolysis Intratissue (EPI^®^): Study of a Population Series of Cases in Sport. Ph.D. Thesis.

[2] Abat F., Valles S.L., Gelber P.E., Polidori F., Jorda A., García-Herreros S., Monllau J.C., Sanchez-Ibáñez J.M. (2015). An Experimental Study of Muscular Injury Repair in a Mouse Model of Notexin-Induced Lesion with EPI^®^ Technique. BMC Sports Sci. Med. Rehabil..

[3] Mattiussi G., Moreno C. (2016). Treatment of Proximal Hamstring Tendinopathy Related Sciatic Nerve Entrapment: Presentation of an Ultrasound-Guided “Intratissue Percutaneous Electrolysis” Application. Muscles Ligaments Tendons J..

[4] Margalef R., Valera-Garrido F., Minaya-Muñoz F., Bosque M., Ortiz N., Santafe M.M. (2020). Percutaneous Needle Electrolysis Reverses Neurographic Signs of Nerve Entrapment by Induced Fibrosis in Mice. Evid.-Based Complement. Altern. Med..

[5] Colmena C. (2013). New Technique in Tendon Sport Recovery. Percutaneous Electrolysis Intratissue (EPI^®^). Int. J. Phys. Med. R. habil..

[6] Zhao M. (2009). Electrical Fields in Wound Healing-An Overriding Signal That Directs Cell Migration. Semin. Cell Dev. Biol..

[7] Rodríguez-Huguet M., Góngora-Rodríguez J., Rodríguez-Huguet P., Ibañez-Vera A.J., Rodríguez-Almagro D., Martín-Valero R., Díaz-Fernández Á., Lomas-Vega R. (2020). Effectiveness of percutaneous electrolysis in supraspinatus tendinopathy: A single-blinded randomized controlled trial. J. Clin. Med..

[8] Abat F., Gelber P.E., Polidori F., Monllau J.C., Sanchez-Ibañez J.M. (2015). Clinica Results after Ultrasound-Guided Intratissue Percutaneous Electrolysis (EPI^®^) and Eccentric Exercise in the Treatment of Patellar Tendinopathy. Knee Surg. Sport. Traumatol. Arthrosc..

[9] García Naranjo J., Barroso Rosa S., Loro Ferrer J.F., Limiñana Cañal J.M., Suarez Hernández E. (2017). A Novel Approach in the Treatment of Acute Whiplash Syndrome: Ultrasound-Guided Needle Percutaneous Electrolysis. A Randomized Controlled Trial. Orthop. Traumatol. Surg. Res..

[10] Gómez-Chiguano G.F., Navarro-Santana M.J., Cleland J.A., Arias-Buría J.L., Fernández-de-Las-Peñas C., Ortega-Santiago R., Plaza-Manzano G. (2021). Effectiveness of Ultrasound-Guided Percutaneous Electrolysis for Musculoskeletal Pain: A Systematic Review and Meta-Analysis. Pain Med..

[11] Carvajal O., Alvarez D., Medina F., Minaya F. (2016). Effects of Percutaneous Needle Electrolysis of the Patellar Tendon on Local and Contralateral Temperature, Measured with Infrared Thermography. Rev. Fisioter. Invasiva.

[12] Margalef R., Bosque M., Minaya-Muñoz F., Valera-Garrido F., Santafe M.M. (2021). Safety Analysis of Percutaneous Needle Electrolysis: A Study of Needle Composition, Morphology, and Electrical Resistance. Acupunct. Med..

[13] Rodríguez-Sanz J., López-de-Celis C., Hidalgo-García C., González-Rueda V., Ragazzi P., Bueno-Gracia E., Llurda-Almuzara L., Pérez-Bellmunt A. (2022). Is tecar therapy effective on biceps femoris and quadriceps rehabilitation? a cadaveric study. J. Sport Rehabil..

[14] Pérez-Bellmunt A., Caballé-Serrano J., Rodríguez-Sanz J., Hidalgo-García C., González-Rueda V., Gassó-Villarejo S., Zegarra-Chávez D., López-de-Celis C. (2022). Comparison of resistive capacitive energy transfer therapy on cadaveric molars and incisors with and without implants. Sci. Rep..

[15] McDaniels A., Pittman D., Cotter A. (2012). Recommendations for Best Needling Practices with Respect to Skin Preparation. Med. Acupunct..

[16] Standring S. (2008). Gray’s Anatomy.

[17] Xian Z., Ali S. (2021). Crossed Doubled Patellar Tendon: A Rare Anatomical Variant with Potential Clinical Significance. Radiol. Case Rep..

[18] Macchi V., Stocco E., Stecco C., Belluzzi E., Favero M., Porzionato A., De Caro R. (2018). The Infrapatellar Fat Pad and the Synovial Membrane: An Anatomo-Functional Unit. J. Anat..

[19] Pierce C., Block R., Aguinis H. (2004). Cautionary Note on Reporting Eta-Squared Values from Multifactor ANOVA Designs. Educ. Psychol. Meas..

[20] Bryant Jeri L., Boughter John D., Gong S., LeDoux Mark S.H.D.H. (2010). Current Body Composition Measurement Techniques. Physiol. Behav..

[21] Rutkove S.B., Sanchez B. (2019). Electrical Impedance Methods in Neuromuscular Assessment: An Overview. Cold Spring Harb. Perspect. Med..

[22] Lopez-Martos R., Gonzalez-Perez L.M., Ruiz-Canela-Mendez P., Urresti-Lopez F.J., Gutierrez-Perez J.L., Infante-Cossio P. (2018). Randomized, Double-Blind Study Comparing Percutaneous Electrolysis and Dry Needling for the Management of Temporomandibular Myofascial Pain. Med. Oral Patol. Oral Cir. Bucal.

[23] Varela-Rodríguez S., Sánchez-Sánchez J.L., Velasco E., Delicado-Miralles M., Sánchez-González J.L. (2022). Endogenous Pain Modulation in Response to a Single Session of Percutaneous Electrolysis in Healthy Population: A Double-Blinded Randomized Clinical Trial. J. Clin. Med..

[24] Peñin-Franch A., García-Vidal J.A., Martínez C.M., Escolar-Reina P., Martínez-Ojeda R.M., Gómez A.I., Bueno J.M., Minaya-Muñoz F., Valera-Garrido F., Medina-Mirapeix F. (2022). Galvanic Current Activates the NLRP3 Inflammasome to Promote Type I Collagen Production in Tendon. Elife.

[25] Wemyss-Holden S.A., Berry D.P., Robertson G.S.M., Dennison A.R., Hall P.D.L.M., Maddern G.J. (2002). Electrolytic Ablation as an Adjunct to Liver Resection: Safety and Efficacy in Patients. ANZ J. Surg..

